# Editorial: Beyond Probiotics: Dietary Microbial Modulators of the Immune System - Effects and Mechanisms

**DOI:** 10.3389/fnut.2022.852086

**Published:** 2022-03-03

**Authors:** Margarida Castell, Gemma E. Walton, Francisco J. Pérez-Cano

**Affiliations:** ^1^Physiology Section, Department of Biochemistry and Physiology, Faculty of Pharmacy and Food Science, University of Barcelona (UB), Barcelona, Spain; ^2^Institute of Research in Nutrition and Food Safety (INSA), University of Barcelona (UB), Santa Coloma de Gramenet, Spain; ^3^Department of Food and Nutritional Sciences, University of Reading, Reading, United Kingdom

**Keywords:** probiotics, prebiotics, postbiotics, SCFA, microbiota, oligosaccharides, metabolites, immune response

In recent years influential foods have emerged with force in the field of immunonutrition, with examples that include pro, pre, and postbiotics and all their combinations receiving particular attention. Probiotics are live microorganisms that, when administered in adequate amounts, confer health benefits on the host and, among others, the immune system. After ingestion, their effects are mediated through modulation of the resident microbiota composition, bioactive molecule production, and changes in epithelial and immune intestinal cells (1). Additionally, prebiotics promote the selective growth of certain beneficial bacteria and can be used in combination with probiotic strains (synbiotics) (1). Besides the direct effect of these compounds on bacterial composition and functionality, they can also impact the immune system and host defense mechanisms through direct interaction with pathogens or with intestinal or immune cells. In recent years, a new “biotic” type has been proposed, postbiotics. This concept refers to the “preparation of inanimate microorganisms and/or their components that confers a health benefit on the host” (2). The goal of this Research Topic was to present the current state-of-the-art research on the effects of microbial modulators, also known globally as “biotics,” on immune function.

This Research Topic gathers six review papers, including two minireviews, four original research papers, and one including hypothesis and theory. Among the interesting reviews included here, the paper of Liu et al. deals with probiotics, prebiotics, and postbiotics. The authors compile the effects of the three types of “biotics” on the gut microbiota and immune function, including clinical and preclinical studies demonstrating their effects and concluding that the mechanisms involved in such effects deserve further studies. Furthermore, Teame et al. introduce the concept of paraprobiotic defining the cell structural components of probiotic bacteria, to differentiate from postbiotics, i.e., metabolites of probiotics. The authors reviewed the effects of paraprobiotics and postbiotics derived from *Lactobacillus*, suggesting their potential as immunomodulators and highlighting them as a valid and safer alternative to live probiotic bacteria. In the same sense, a review concerning the role of low-doses of lactulose as a prebiotic is also included in this collection (Karakan et al.). The authors compile clinical and preclinical studies about the effects of such compounds in the composition and metabolites of the gut microbiota as well as calcium absorption. With regard to the fourth review, Rohrhofer et al. focused on the effects of dietary bioactive sphingolipids on the gut microbiota and the intestinal barrier, concluding that such compounds could combat chronic, low-grade intestinal inflammation and subsequent metabolic diseases.

From a different point of view, the paper of De Juan et al. is a minireview that focuses on a possible mechanism of action for microbiota metabolites. Such compounds could be ligands for the aryl hydrocarbon receptor (AhR), which can act as a transcription factor that induces anti-inflammatory and immunoregulatory effects.

On the other hand, the minireview of Liu et al. summarizes the relationship between the gut and respiratory microbiota and tuberculosis and discusses the role of “biotics” in the treatment of respiratory disease. The paper evidences the importance of the gut-lung axis and that it should be considered in the treatment of respiratory diseases. In a similar point of view, the paper of Yang et al. hypothesizes the importance of a healthy microbiota in people with diabetes mellitus. The authors hypothesize about the association between low levels of microbial short-chain fatty acids (SCFA) and gut dysbiosis in diabetic people, with inflammatory responses and ulterior tumorigenesis.

The Research Topic includes four interesting research articles. One of them focuses on probiotics, in particular, the effects of *Lacticaseibacillus rhamnosus* CRL1505 in a mouse model of respiratory syncytial virus infection (Garcia-Castillo et al.). Using this model, the authors hypothesize the mechanism of action of the probiotic as involving alveolar macrophages and CD4+ cells as well interferon γ production modulation.

Two of the research articles included in this Research Topic refer to prebiotic effects. Thus, Xu et al. demonstrate that polysaccharides from a mushroom, *Tremella fuciformis*, could prevent a colonic inflammation induced in mice. The authors consider the mechanisms involved in such protective effects to be driven by changes in the intestinal microbiota. Another preclinical study demonstrated the protective effect of a prebiotic from citrus pulp against the weaning stress of piglets (Uerling et al.). The authors showed the effects of this prebiotic in the intestinal health of piglets as well as the influence on their microbiota.

Finally, a promising clinical study was carried out in Uganda by comparing the effects of the administration of probiotic yogurt or that of milk on the incidence rate for common cold symptoms and the skin infection symptoms of children aged 3 to 6 years old (Westerik et al.). Although the authors recognize that the study had some limitations, they observed a positive trend of yogurt against the incidence of common cold and skin infections, therefore opening up new perspectives for further studies.

This Research Topic includes interesting papers focusing on “biotics” from different types and origins, demonstrating that a large number of products can be included in this category. In addition, overall, they highlight the role of “biotics” not only in intestinal health but also in respiratory and cutaneous infections. Further studies are encouraged to expand the knowledge of these bioactive compounds, to establish their modes of action, and above all to demonstrate, by means of clinical studies, the importance of such products in health.

## Author Contributions

All authors contributed equally to the article and approved the submitted version.

## Conflict of Interest

The authors declare that the research was conducted in the absence of any commercial or financial relationships that could be construed as a potential conflict of interest.

## Publisher's Note

All claims expressed in this article are solely those of the authors and do not necessarily represent those of their affiliated organizations, or those of the publisher, the editors and the reviewers. Any product that may be evaluated in this article, or claim that may be made by its manufacturer, is not guaranteed or endorsed by the publisher.
